# Speed and Convenience Aren't Everything with Diagnostics

**DOI:** 10.1371/journal.pmed.1001113

**Published:** 2011-10-25

**Authors:** The PLoS Medicine Editors

## Abstract

The *PLoS Medicine* editors reflect on recent research and analysis that highlight the need to fully evaluate rapid diagnostics.

In an age when we can sequence an entire genome within a day, we expect to be able to gather and access accurate information at a pace. After all, you didn't wait for this issue of *PLoS Medicine* to arrive in the post or trudge down to the library to read it; you accessed it instantly via the internet. Patients and clinicians also expect technology to provide similar speed when it comes to diagnosing infectious disease. Recent research and analysis published in *PLoS Medicine* reveals a theme of rapid diagnostics and raises the question of whether providing an answer quickly is enough to produce meaningful health outcomes.

For patients suffering from any infectious disease, diagnosis should pave the way to treatment. Reducing the lag time between testing and diagnosis has obvious advantages for the patient by ensuring timely receipt of care, and this should in turn benefit others by reducing the probability of transmission. In December last year the World Health Organization (WHO) endorsed the use of a new automated PCR-based test for tuberculosis (TB), known as Xpert, which can rapidly confirm infection and detect resistance to rifampicin [1],[2]. Many countries currently rely on sputum smear microscopy and culture to diagnose TB, which can take weeks to provide results. The new test takes under two hours to provide a diagnosis, fueling high hopes that it will transform TB diagnosis and therefore treatment. However, there is an additional cost associated with Xpert—each test cartridge currently costs approximately US$17 [3]—and despite being sold at reduced rates in countries where TB is endemic, question marks remain about whether it will be a cost-effective option in low- and middle-income settings.

Since speed and convenience come at a cost, is that cost worthwhile? In a recent *PLoS Medicine* essay, David Dowdy and colleagues highlighted the challenges of determining the cost-effectiveness of rapid TB diagnostics, including the danger of draining resources from other TB-specific interventions and the need to take into account the cost of treating false-positive diagnoses [4]. WHO's recent policy recommendation against the use of rapid commercial serological tests for active TB serves as a stark reminder of the need to evaluate diagnostics fully [5]. The kits may be both rapid and convenient, but as a meta-analysis by Karen Steingart and colleagues in *PLoS Medicine* has shown, they fail in their primary purpose since they are neither accurate nor consistent enough to replace sputum smear microscopy as a test for TB [6]. The consequences of this failure have been substantial because the kits are widely used in countries with the highest TB burden, and it has been suggested that the cost of testing and treating false-positives may rival the annual budget of India's entire TB control program ($65 million) [7]. While the accuracy of Xpert is not in doubt, substantial challenges remain before the scale-up of this rapid test delivers on its promise.

Speedier and more convenient still are diagnostics that can be packaged into reliable self-test kits, which may improve testing uptake for diseases that carry a significant social stigma, such as HIV. Anthony Choko and colleagues report in *PLoS Medicine* that self-testing for HIV can be used in the field to produce accurate results and may indeed improve testing uptake [8]. In their study, just over 90% of people interviewed agreed to test for HIV using an oral self-test kit, with most expressing a preference for self-testing for future HIV tests. Particularly encouraging is the high proportion of men reporting a preference to be self-tested in this way, as they are a demographic who in this setting have a poor record for HIV testing.

However, an important question remains unanswered by this research: is there any advantage to a 20 minute test if a self-diagnosed person remains isolated from counseling or care? This is a question raised in an illuminating Perspective article by Rochelle Walensky and Ingrid Bassett that accompanies the new study [9]. They argue that knowing one's HIV status is undoubtedly a critical first step, but that we must ensure that increased speed and convenience of testing doesn't break the chain of care. If we can't ensure that rapid testing is translated into care we are in danger of going nowhere fast.

We are fortunate to live in a time when technology enables us to rapidly gather and process information; the ability to do so is transforming health care. However, these papers serve as a timely reminder that rapidly detecting the cause of an illness is not in itself enough to significantly change health outcomes.

## Abbreviations

TB: tuberculosis
WHO: World Health Organization
